# Macromolecular crowding meets oxygen tension in human mesenchymal stem cell culture - A step closer to physiologically relevant *in vitro* organogenesis

**DOI:** 10.1038/srep30746

**Published:** 2016-08-01

**Authors:** Daniela Cigognini, Diana Gaspar, Pramod Kumar, Abhigyan Satyam, Senthilkumar Alagesan, Clara Sanz-Nogués, Matthew Griffin, Timothy O’Brien, Abhay Pandit, Dimitrios I. Zeugolis

**Affiliations:** 1 Regenerative, Modular & Developmental Engineering Laboratory (REMODEL), Biosciences Research Building, National University of Ireland Galway (NUI Galway), Galway, Ireland; 2 Science Foundation Ireland (SFI) Centre for Research in Medical Devices (CÚRAM), Biomedical Sciences Building, NUI Galway, Galway, Ireland; 3 Regenerative Medicine Institute (REMEDI), Biomedical Sciences Building, NUI Galway, Galway, Ireland

## Abstract

Modular tissue engineering is based on the cells’ innate ability to create bottom-up supramolecular assemblies with efficiency and efficacy still unmatched by man-made devices. Although the regenerative potential of such tissue substitutes has been documented in preclinical and clinical setting, the prolonged culture time required to develop an implantable device is associated with phenotypic drift and/or cell senescence. Herein, we demonstrate that macromolecular crowding significantly enhances extracellular matrix deposition in human bone marrow mesenchymal stem cell culture at both 20% and 2% oxygen tension. Although hypoxia inducible factor - 1*α* was activated at 2% oxygen tension, increased extracellular matrix synthesis was not observed. The expression of surface markers and transcription factors was not affected as a function of oxygen tension and macromolecular crowding. The multilineage potential was also maintained, albeit adipogenic differentiation was significantly reduced in low oxygen tension cultures, chondrogenic differentiation was significantly increased in macromolecularly crowded cultures and osteogenic differentiation was not affected as a function of oxygen tension and macromolecular crowding. Collectively, these data pave the way for the development of bottom-up tissue equivalents based on physiologically relevant developmental processes.

Current tissue engineering and regenerative medicine therapies are primarily focused on direct cell injections or are utilising a carrier system. However, cell injections are associated with poor cell localisation at the side of injury (within hours post-implantation) and carrier-based approaches are frequently accompanied by foreign body/immune responses (within days post-implantation). To overcome these limitations, modular tissue engineering has emerged, during which cells produce their own carrier (extracellular matrix; ECM), which in turn enhances cell localisation at the side of injury. The clinical relevance/potential of such cell-assembled systems has already been documented for skin^1^, cornea^2^ and blood vessel^3^, whilst very promising preclinical data have been demonstrated for heart^4^, lung^5^, bone^6^ and liver^7^. Nonetheless, the rate limiting factor for wide acceptance of this physiologically relevant technology is the prolonged culture time required to develop an implantable device (e.g. 196 days for blood vessel^8^), which is associated with phenotypic drift, cell senescence and consequently loss of cells’ therapeutic potential. To this end, numerous *in vitro* microenvironment modulators are at the forefront of scientific and technological research and innovation to either direct stem cells towards a specific lineage or to maintain stem cells’ and permanently differentiated cells’ phenotype during *ex vivo* expansion^9^,^10^,^11^,^12^. In particular, the ability to maintain stem cell function during *ex vivo* growth is fundamental for the development of reparative therapies, as their bioactive, trophic, immunomodulatory, angiogenic and anti-apoptotic secretome determines their therapeutic efficacy^13^,^14^,^15^.

Given the complexity of the *in vivo* milieu, recent data advocate that multifactorial cell expansion approaches are likely to lead in clinically relevant cell therapies^16^,^17^,^18^,^19^. Among the various methods of microenvironmental induced signalling, physiological low oxygen tension has been shown to be of the utmost importance in maintaining stem cell phenotype, controlling their differentiation and fate and increasing their motility and therapeutic potential^20^,^21^,^22^,^23^. Further, through the activation of hypoxia inducible factor - 1*α* (HIF-1*α*), cell metabolism is regulated^24^, cell cycle quiescence is maintained^25^, angiogenesis is promoted^26^ and ECM synthesis is enhanced^27^,^28^, which in turn has been shown to be crucial in regulating stem cell fate^29^,^30^. However, in the physiologically irrelevant dilute culture media, this *de novo* synthesised ECM is dispersed and discarded during media changes. We have recently demonstrated that the addition of inert and polydispersed macromolecules in culture media [e.g. carrageenan (galactose-based) 550 kDa (estimated); dextran sulphate (glucose) 500 kDa; Ficoll^™^ (sucrose) cocktail of 70 kDa and 400 kDa] not only accelerates by up to 80-fold ECM deposition, but also maintains permanently differentiated cell phenotype, even at low density and low serum cultures^31^,^32^,^33^. This was attributed to macromolecules crowding (MMC)/excluding volume effect, a biophysical phenomenon that governs the physiological environment of multicellular organisms and intensifies biological processes and thermodynamic rates by several orders of magnitude^34^,^35^. In a sense, MMC, by imitating the dense and confined native tissue context, accelerates biological processes, such as the enzymatic conversion of procollagen to collagen^36^,^37^,^38^, which is onerous in the customarily used dilute culture conditions. To-date, although MMC has been shown to enhance and to organise ECM deposition in naïve stem cell culture^39^,^40^,^41^ and to enhance adipogenesis in adipose-induced stem cells^42^, its influence on naïve stem cell phenotype has yet to be demonstrated. Further, no study has assessed the simultaneous effect of MMC and oxygen tension in the development of tissue-like supramolecular assemblies. Herein, we ventured to assess the synergistic effect of oxygen tension and MMC in human bone marrow mesenchymal stem cell (hBMSC) culture (Fig. 1).

## Materials and Methods

### Cell culture

hBMSCs were isolated (see Supporting Information) from fresh bone marrow (Lonza) and cultured in alpha - Minimum Essential Medium with GlutaMAX^™^ (Gibco Life Technologies) supplemented with 10% Hyclone^™^ foetal bovine serum (Thermo Scientific) and 1% penicillin-streptomycin at 37 °C in a humidified atmosphere of 5% CO_2_. At passages 2–4, cells were seeded at 25,000 cells/cm^2^ in 24 well plates and were allowed to attach for 24 hours. After 24 hours, medium was changed to medium with MMC [CR at 1, 5, 10, 50, 100 and 500 *μ*g/ml (Sigma) or FC [37.5 mg/ml of Ficoll™ 70 (Sigma Aldrich) and 25 mg/ml of Ficoll™ 400 (Sigma Aldrich)]. Supplementation with 100 *μ*M of L-ascorbic acid phosphate (Sigma Aldrich) was used to induce collagen synthesis. Medium with MMC was changed every 3 days. For the low oxygen tension experiments, cells were maintained in the Oxygen Tissue Culture Glove Box (Coy Lab Products) at 2% O_2_ and 37 °C in a humidified atmosphere.

### Collagen extraction

At days 2, 4, 7 and 14, culture medium was collected and cell layers were digested with porcine gastric mucosa pepsin (Sigma Aldrich) at a final concentration of 0.1 mg/ml in 0.05 M acetic acid. Cell layers were incubated for 2 hours at 37 °C with gentle shaking, after which they were neutralised with 0.1 N NaOH.

### SDS-PAGE and densitometric analysis

Medium and cell layer were analysed by SDS-PAGE under non-reducing conditions with Mini-Protean^®^ 3 electrophoresis system (Bio-Rad Laboratories). Bovine collagen type I (Symatese Biomateriaux) was used as control in every gel. Protein bands were stained with the SilverQuest™ kit (Invitrogen) according to the manufacturer’s protocol. Densitometric analysis of the *α*1 and *α*2 bands was performed with ImageJ software (NIH).

### Gelatine zymography analysis

Culture medium was collected and cell layers were lysed and scrapped in 1x Laemmli buffer (Sigma Aldrich). Samples were analysed using 10% SDS-PAGE gels containing 1 mg/ml of gelatine (Sigma Aldrich). After electrophoresis, gels were placed in a washing buffer (2.5% Triton X-100, 50 mM Tris Base, 5 mM CaCl_2_, 1 *μ*M ZnCl_2_; all Sigma Aldrich) for 1 hour and washed once with water. Gels were then incubated for 16–20 hours at 37 °C in reaction buffer (50 mM Tris Base, 5 mM CaCl_2_, 1 *μ*M ZnCl_2_). Staining was performed with 0.5% Coomassie Brilliant Blue (Sigma Aldrich) in 30% ethanol and 10% acetic acid for 20 minutes and de-stained for 2 hours.

### Immunocytochemistry analysis

Cells were seeded on 4 or 8 well Lab-Tek^™^ II chamber slides (Nunc, Thermo Scientific) at a density of 25,000 cells/cm^2^. At each time point cells were washed with phosphate buffered saline (PBS, Sigma Aldrich) and fixed in 4% paraformaldehyde (Sigma Aldrich). For intracellular epitopes, cells were permeabilised with 0.1% Triton in PBS for 10 minutes. Non-specific sites were blocked with 3% bovine serum albumin (Sigma Aldrich) for 1 hour, then cells were incubated overnight at 4 °C with primary antibodies for collagen type I (Abcam, 90395), collagen type III (Abcam, ab7778), fibronectin (Sigma Aldrich, F7387), laminin (Sigma Aldrich, L9393) and HIF-1*α* (Abcam, ab113642). Bound antibodies were visualised after incubation with AlexaFluor^®^ 488 goat anti-mouse or AlexaFluor^®^ 568 goat anti-rabbit (Life Technologies) secondary antibodies for 45 minutes. Cell nuclei were counterstained with 4,6-diamidino-2-phenylindole (DAPI, Life Technologies). Slides were mounted with FluorSave^™^ (Merck Millipore). Images were acquired using an inverted fluorescence microscope (Olympus IX-81) and analysed with ImageJ software.

### Flow cytometry analysis

Cells were suspended in FACS buffer at a concentration of 5 × 10^6^/ml. For surface marker staining, cells were incubated with various combinations of fluorochrome-labeled antibodies (CD90, CD44, CD105, CD73 along with isotype controls) according to manufacturer’s instructions (BD Stemflow^™^). For intracellular staining, cells were washed with Cytofix/Cytoperm Plus^®^ (BD Biosciences) and stained for transcriptional factors OCT-4 (clone C30A3, rabbit mAb Alexa Fluor 488 Conjugate, Cell Signaling Technology, 5177S), SOX-2 (clone D6D9, XP^®^ rabbit mAb Alexa Fluor 488 Conjugate, Cell Signaling Technology, 5046S), SSEA-4 (clone MC813-70, mouse mAb PE conjugate, BD Biosciences, 560128) and Nanog (clone D73G4, XP^®^ rabbit mAb Alexa Fluor 647 Conjugate, Cell Signaling Technology, 5448S) for 30 minutes at 4 °C. Cells were analysed using a FACSCanto^®^ cytometer (BD Biosciences). Median Fluorescence Intensity (MFI) of hBMSCs was calculated using FlowJo^®^ software (TreeStar Inc.) and fold increase over the appropriate isotype control was averaged.

### Gene expression analysis

Total RNA was extracted and purified using RNeasy^®^ Mini Kit (Qiagen), following manufacturer’s protocol. DNA was removed during RNA purification using RNase-Free DNase Set (Quiagen). RNA quality and quantity were analysed on the Agilent 2100 Bioanalyser (Agilent Technologies). Reverse transcription of extracted mRNA was performed using the ImProm-II^™^ Reverse Transcription System (Promega). Real-Time PCR was performed using StepOnePlus^™^ instrument (Applied Biosystems^®^, Life Technologies) and data were analysed with StepOne Software. The 25 *μ*l reaction contained Quanti Fast SYBR Green (Quiagen), primers in a final concentration of 0.2 *μ*M each and 12.5 ng cDNA. The expression of target genes was normalised to the geometric mean of the expression of *β*-actin and cyclophilin, which were used as reference genes. The expression (or fold change) of the target gene relative to the control (cells maintained in CTR medium at 20% O_2_ at day 7) was calculated with the 2^−∆∆Ct^ method. Primers were designed using Primer-BLAST (http://blast.ncbi.nlm.nih.gov/Blast.cgi) and they were analysed using OligoAnalyzer 3.1 (http://eu.idtdna.com/calc/analyzer) and Primer3 (http://bioinfo.ut.ee/primer3-0.4.0). Primers used can be found in Table S1.

### Statistical analysis

Unless otherwise stated, each assay was repeated in three independent experiments and each experiment was performed in triplicate. Data were processed using GraphPad Prism^®^ 5 software and reported as mean ± standard deviation. Comparisons among groups were performed by one-way ANOVA, followed by Bonferroni’s multiple comparison test. One-way ANOVA was employed after confirming the following assumptions: (a) the distribution of the sample mean was normal (Anderson-Darling normality test); and (b) the variances of the population of the samples were equal to one another (Bartlett’s and Levene’s tests for homogeneity of variance). Kruskal-Wallis test, followed by *Dunn’s* multiple comparison test, was used when either or both of the above assumptions were violated. Statistical significance was accepted at *p* < 0.05.

## Results

**Figure 2:**
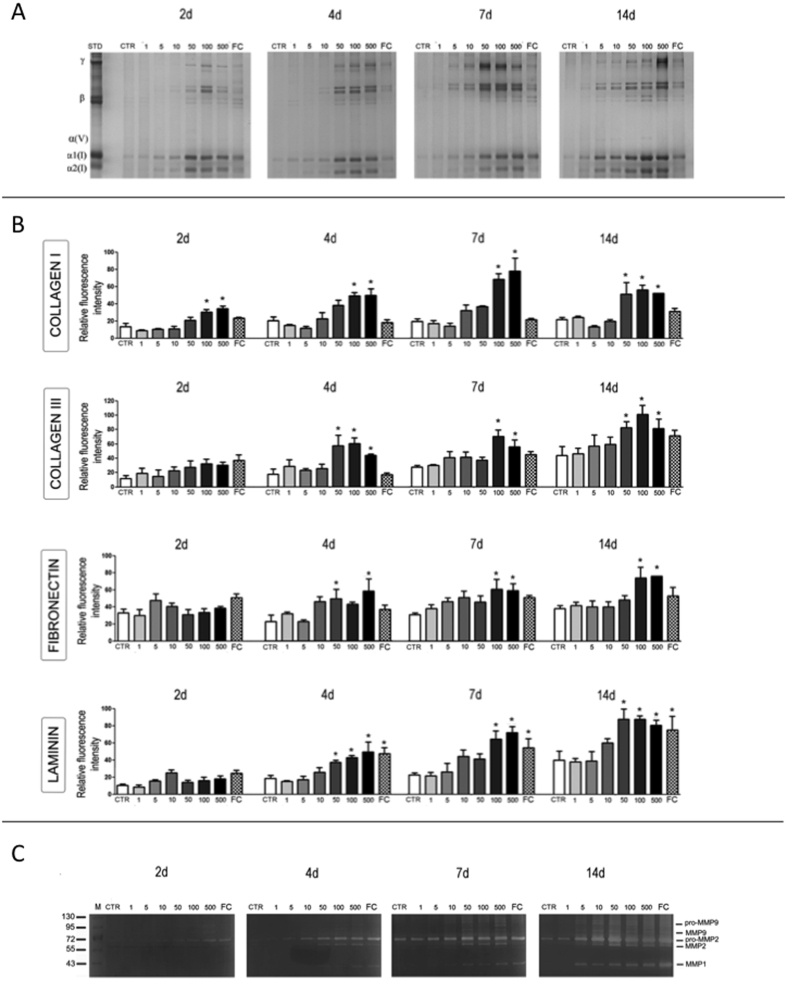
(**A**) SDS-PAGE analysis of cell layers at 2, 4, 7 and 14 days indicates that 100 *μ*g/ml CR is the minimum effective concentration of CR for maximum ECM deposition. (**B**) Relative fluorescence intensity of immunofluorescence analysis for collagen type I, collagen type III, fibronectin and laminin further corroborates the observation that 100 *μ*g/ml CR is the minimum effective concentration of CR for maximum ECM deposition. (**C**) Gelatine zymography of the cell layers reveals an increased MMP activity as a function of CR concentration. *Statistical significance from CTR at a given time point.

To assess the influence of MMC on ECM deposition, different concentrations of carrageenan (CR) were assessed and compared to the Ficoll™ cocktail (FC) and the non-MMC control (CTR). Collagen type I deposition was assessed by sodium dodecyl sulphate-polyacrylamide gel electrophoresis (SDS-PAGE; Fig. 2A) and corresponding densitometric analysis (Figure S1) revealed that CR concentrations in the range of 50 to 500 *μ*g/ml induced the highest (*p* < 0.001) collagen type I deposition at all time points assessed (2, 4, 7 and 14 days). This enhanced collagen type I deposition in the cell layer at the effective CR concentrations (50 and 500 *μ*g/ml) was accompanied by a decrease in collagen concentration in the media, as revealed by SDS-PAGE and complementary densitometric analysis (Figure S2). Immunocytochemistry (Figures S3 and S4) and complementary relative fluorescence intensity (Fig. 2B) analyses demonstrated significantly enhanced (*p* < 0.001) collagen type I, collagen type III, fibronectin and laminin deposition in the presence of 100 and 500 *μ*g/ml CR after 4, 7 and 14 days in culture. Gelatine zymography of the cell layers revealed an increased matrix metalloproteinase (MMP) activity as a function of CR concentration and, by day 14, MMP activity was significantly increased (*p* < 0.001) for all but one (1 *μ*g/ml CR) MMC groups (Fig. 2C). Gelatin zymography of the media (Figure S5) revealed an increased pro-MMP2 activity as a function of time in culture (*p* < 0.001), but no difference was detected among the groups (*p* > 0.05). By day 14, pro-MMP9 was also detected in all groups (Figure S5). Cell metabolic activity (Figure S6A), viability (Figure S6B) and morphology (Figure S6C) were not affected, independently of the treatment and time in culture.

**Figure 3:**
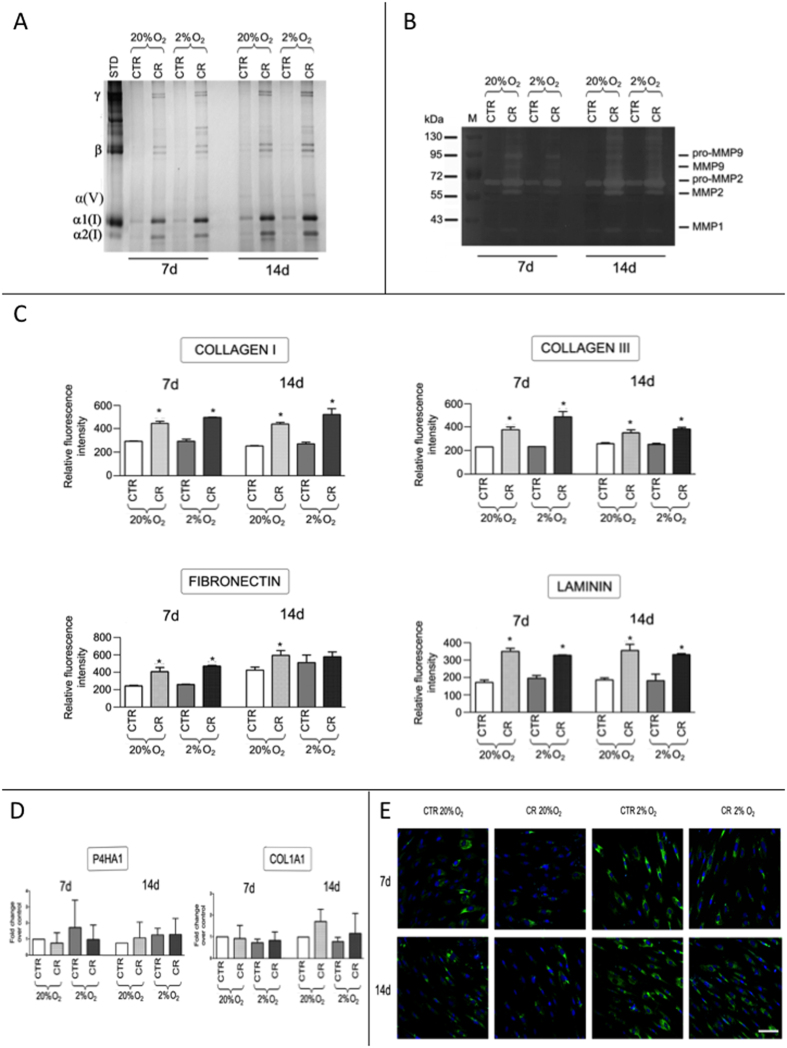
(**A**) SDS-PAGE analysis of the cell layers indicates a significantly higher ECM deposition under MMC conditions at both oxygen tensions (20% and 2%) and time points (7 and 14 days), but no significant difference is apparent at low oxygen tension cultures. (**B**) Gelatin zymography of the cell layers reveals a significantly higher MMP activity under MMC conditions at both oxygen tensions (20% and 2%) and time points (7 and 14 days). (**C**) Relative fluorescence intensity of immunofluorescence analysis for collagen type I, collagen type III, fibronectin and laminin further corroborates the observation that MMC enhances ECM deposition at both oxygen tensions (20% and 2%) and time points (7 and 14 days). (**D**) Relative gene expression analysis of *α*1(I) procollagen and P4HA1 reveals no significant difference in their expression as a function of MMC and oxygen tension at both time points. (**E**) Immunocytochemistry analysis of HIF-1*α* reveals no significant difference between the MMC and non-MMC groups at both time points (7 and 14 days), whilst significantly higher HIF-1*α* expression is detectable for cells grown at 2% oxygen tension. Scale bar: 100 *μ*m. *Statistical significance from CTR at a given time point and oxygen tension.

To assess the simultaneous effect of oxygen tension and MMC on ECM deposition, hBMSCs were cultured at 20% and 2% oxygen tension in the absence (CTR) and presence of 100 *μ*g/ml CR. SDS-PAGE of the cell layers (Fig. 3A) revealed a significantly higher (*p* < 0.001) ECM deposition between the CR and CTR groups at both oxygen tensions (20% and 2%) and time points (7 and 14 days), but no significant difference (*p* > 0.05) was observed in collagen type I deposition between the MMC and non-MMC groups at 20% and 2% oxygen tension and both time points (7 and 14 days). Similarly, gelatin zymography of the cell layers (Fig. 3B) demonstrated a significantly higher (*p* < 0.001) MMP activity between the CR and CTR groups at both oxygen tensions (20% and 2%) and time points (7 and 14 days), but no significant difference (*p* > 0.05) was observed in MMP activity between the MMC and non-MMC groups at 20% and 2% oxygen tension and both time points (7 and 14 days). Immunocytochemistry (Figures S7 and S8) and complementary relative fluorescence intensity (Fig. 3C) analyses further corroborated these observations, as a significantly higher (*p* < 0.001) collagen type I, collagen type III, fibronectin and laminin deposition was observed between the CR and CTR groups at both oxygen tensions (20% and 2%) and time points (7 and 14 days), but no significant difference (*p* > 0.05) in their deposition was observed between the MMC and the non-MMC counterparts at 20% and 2% oxygen tension and both time points (7 and 14 days). Relative gene expression analysis (Fig. 3D) of *α*1(I) procollagen and prolyl 4 hydroxylase 1 (P4HA1) revealed no significant difference (*p* > 0.05) in their expression, independently of the presence or absence of CR and oxygen tension (20% and 2%) at both time points (7 and 14 days). In contrast, immunocytochemistry analysis of HIF-1*α* (Fig. 3E) revealed no significant difference between the MMC and non-MMC groups at both time points (7 and 14 days), whilst significantly higher (*p* < 0.001) HIF-1*α* expression (Fig. 3E) was detected for cells grown at 2% oxygen tension, independently of the presence or absence of CR. Cell metabolic activity (Figure S9A) was significantly higher (*p* < 0.001) at 2% oxygen tension, independently of the presence or absence of CR at both time points (7 and 14 days). Cell viability (Figure S9B) and morphology (Figure S9C) were not affected (*p* > 0.05) as a function of oxygen tension (20% and 2%) and presence or absence of CR.

**Figure 4:**
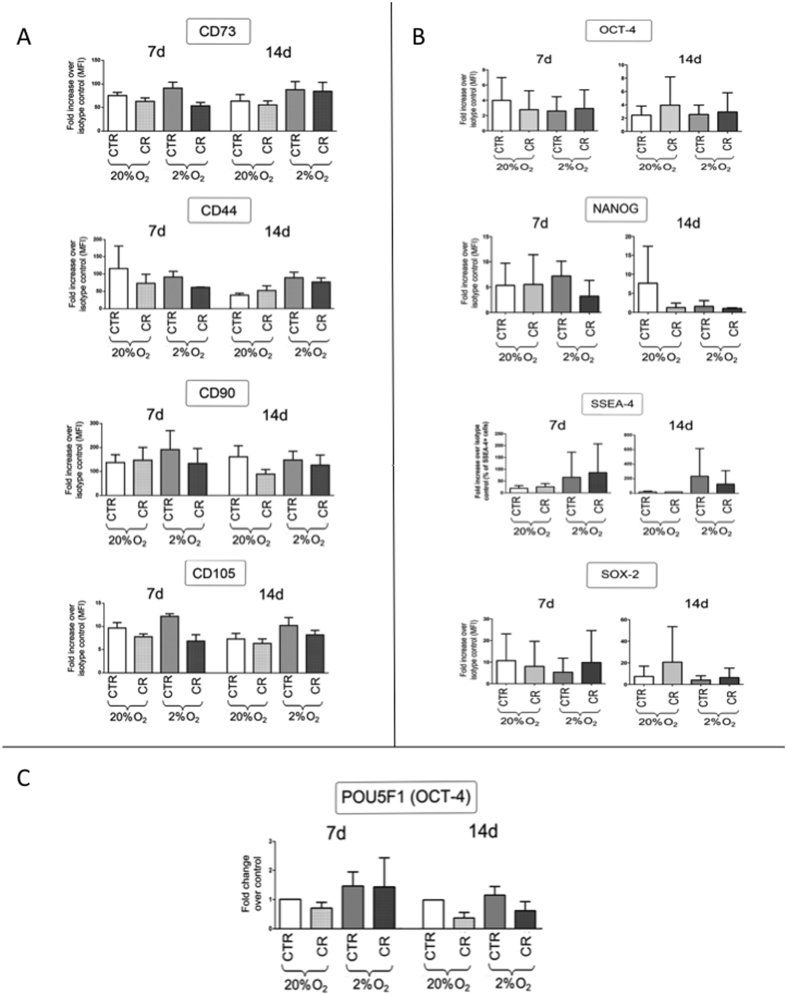
(**A**) FACS analysis indicates no significant difference in surface marker expression as a function of oxygen tension (20% and 2%) and MMC. (**B**) FACS analysis indicates no significant difference in transcriptional factors expression as a function of oxygen tension (20% and 2%) and MMC. (**C**) Relative gene expression analysis shows no significant difference in OCT-4 (POUF-5) expression at both time points (7 and 14 days), independently of the oxygen tension (20% and 2%) and MMC.

To determine whether low oxygen tension and/or MMC influence the multipotent phenotype of hBMSCs, fluorescence activated cell sorting (FACS) analysis was carried out (Figure S10). Surface markers CD90, CD44, CD105 and CD73 were expressed in all groups and no significant difference (*p* > 0.05) was observed independently of the oxygen tension (20% and 2%) and the presence or absence of CR (Fig. 4A). Similarly, no significant difference (*p* > 0.05) was observed in transcriptional OCT-4, SOX-2, Nanog and SSEA-4 expression, independently of the oxygen tension (20% and 2%) and the presence or absence of CR (Fig. 4B). Complementary relative gene expression analysis showed no significant difference (*p* > 0.05) in OCT-4 (POUF-5) expression at both time points (7 and 14 days), independently of the oxygen tension (20% and 2%) and the presence or absence of CR (Fig. 4C), whilst the expression of SOX-2, Nanog and SSEA-4 was too low to be quantified.

**Figure 5:**
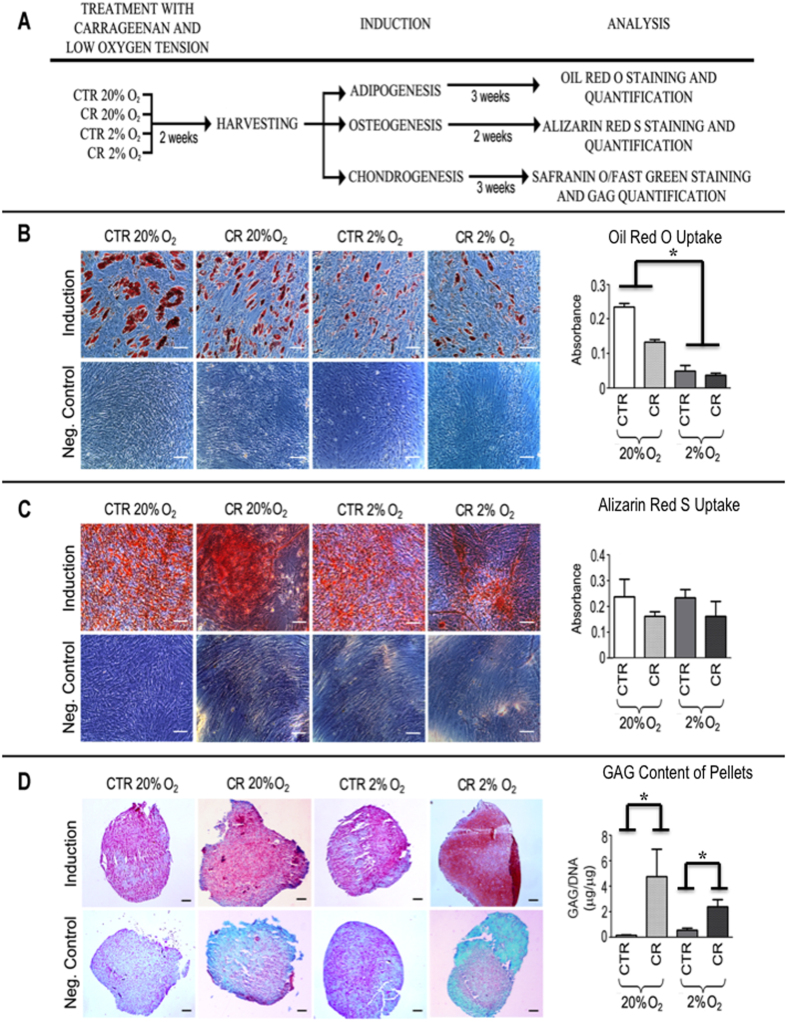
(**A**) Schematic illustration of the experimental design. (**B**) Adipogenic differentiation is significantly reduced in low oxygen tension cultures, independently of the presence or absence of CR. (**C**) Osteogenic differentiation was not affected as a function of oxygen tension and MMC. (**D**) Chondrogenesis was significantly increased under MMC conditions, but no difference was observed between 20% and 2% oxygen tension cultures. Scale bar: 100 *μ*m. * Statistically significant.

The influence of oxygen tension and/or MMC on hBMSCs phenotype was also assessed using tri-lineage differentiation assays (Fig. 5A). Adipogenic differentiation was significantly reduced (*p* < 0.001) in low oxygen tension cultures, independently of the presence or absence of CR (Fig. 5B). All treatments exhibited similar levels (*p* > 0.05) of osteogenic differentiation (Fig. 5C). Chondrogenic differentiation was not supressed as a function of oxygen tension and MMC, with MMC treatments showing significantly higher (*p* < 0.001) glycosaminoglycan (GAG) content at both 20% and 2% oxygen tension (Fig. 5D).

## Discussion

Permanently differentiated and stem cell phenotype maintenance during *in vitro* expansion is at the forefront of scientific research and technological innovation for clinical translation and commercialisation of cell-based therapies. Although significant strides have been achieved using biophysical (e.g. surface topography, substrate rigidity and mechanical loading), biochemical (e.g. media supplements) and biological (e.g. growth factors) signals, the vast number of permutations on, for example, biochemical and/or biological media additives, concentrations, combinations and timings, has restricted the development of clinically relevant and commercially viable therapies. Further, none of these technologies enhances ECM deposition, resulting in prolonged cultures for the development of implantable devices based on the principles of modular tissue engineering, which are often associated with phenotypic drift and loss of cells’ therapeutic potential. Herein, we ventured to assess the simultaneous effect of oxygen tension and MMC on hBMSCs ECM deposition and phenotype maintenance. The rationale of our approach is based on the simplicity in the implementation of the individual elements.

SDS-PAGE and immunocytochemistry analyses clearly demonstrated an enhanced ECM deposition under MMC conditions, which was dependent on crowder present (CR or FC) and CR concentration. In dilute culture media, the *de novo* synthesised water soluble pro-collagen is dissolved before its *N*- and *C*- propeptide extensions are cleaved by the respective proteinases. The addition of inert macromolecules in the culture media restricts pro-collagen and proteinases diffusion, resulting in almost instantaneous pro-peptide extension cleavage and subsequent enhanced ECM deposition^36^,^37^,^38^. The 100 *μ*g/ml CR concentration appeared to be the most effective in inducing maximum ECM deposition. Considering the theory of excluding volume effect, low concentrations of CR allow diffusion of pro-collagen/proteinases in the culture media, thus limiting ECM deposition. The superiority of CR over FC in maximising ECM deposition is attributed to the inherent polydispersity of CR that most effectively excludes volume, as we have demonstrated previously^31^. Of significant importance in the field of *in vitro* organogenesis is the enhanced MMP activity detected in the cell layers, as a function of enhanced ECM deposition, which was induced by the addition of CR, given that MMPs are associated with cell differentiation and migration, ECM maturation and tissue remodelling, angiogenesis and morphogenesis^43^,^44^,^45^,^46^.

Continuing with the simultaneous effect of low oxygen tension and MMC, we observed that MMC once more was effective in increasing ECM deposition. Although immunocytochemistry analysis indicated enhanced HIF-1*α* expression at low oxygen tension, as has been shown before^47^, this was not accompanied by enhanced ECM synthesis and subsequent deposition, as evidenced by SDS-PAGE, immunocytochemistry analysis and gene analysis. Our data are in accordance to previous studies, where adipose derived stem cells grown under low oxygen tension showed reduced ECM synthesis and cytokine type II secretion^48^. However, our data contradict previous studies with differentiated human embryonic stem cells^49^, permanently differentiated cells (e.g. chondrocytes^50^,^51^, fibroblasts^28^, renal epithelial cells^27^) and naïve stem cells^52^,^53^, where enhanced ECM synthesis was observed under low oxygen tension conditions. It is interesting to also note the different impact of low oxygen tension on cells from different regions of intervertebral disc: low oxygen tension increased ECM synthesis in nucleus pulposus cells, whilst low oxygen tension did not bring about any significant difference in ECM synthesis in annulus fibrosus cells^54^. Collectively, these data suggest that activation of HIF-1*α* alone does not necessarily mean increased ECM synthesis; cell-specific endogenous factors (e.g. species; donor age and/or gender; tissue origin; cell differentiation stage) are crucial regulators of the influence of low oxygen tension on ECM synthesis. It is evidenced that further studies are required to elucidate the mechanism underlying the effect of HIF-1*α*, HIF-2*α* and other factors in ECM synthesis of permanently differentiated and stem cell populations. Nonetheless, our data demonstrate that tissue-engineering approaches based on low oxygen tension can be adapted to incorporate MMC without compromising ECM synthesis.

Although it has been well-established in the literature that low oxygen tension (2% to 8%) is a critical factor in maintaining stem cell phenotype, function and self-renewal *ex vivo*^20^,^55^,^56^, customarily *in vitro* cell expansion erroneously takes place at hyperoxic and deleterious for the cells high oxygen tensions (18% to 20%), which are often associated with oxidative stress, DNA damage, growth arrest and loss of cells’ native phenotype and function^57^,^58^. In this study, no significant differences were found in the expression of surface markers and transcriptional factors of hBMSCs, independently of the presence or absence of CR and oxygen tension (20% and 2%). Although osteogenic differentiation was not affected as a function of CR and oxygen tension, low oxygen tension reduced adipogenesis, as evidenced by the presence of less lipid vacuoles. These observations are in agreement with previous studies, where low oxygen tension supressed adipogenesis in a HIF-1*α* dependent manner^59^,^60^, but contradict to previous studies, where osteogenesis was either promoted^59^,^60^ or supressed^61^ in low oxygen tension (<1%) cultures due to downregulation of BMP2 and Runx2 expression^62^ and inhibition of metabolic switch and mitochondrial function^63^. It is also worth noting that extreme low oxygen tension (0.2%) impaired osteogenic differentiation and enhanced adipogenic differentiation through over-expression of HIF-1*α* and CCAAT enhancer-binding proteins^64^. Further, although CR increased GAG content in the pellets, chondrogenesis was not affected as a function of low oxygen tension, which contradicts previous observations, where chondrogenic differentiation was promoted via activation of SOX-9, again, in a HIF-1*α* dependent manner^65^. It is also worth pointing out that previous studies have demonstrated that low oxygen tension cultures diminished chondrogenesis of adipose derived stem cells^66^. It appears that stem cell phenotype maintenance and/or differentiation as a function of oxygen tension, similarly to ECM synthesis as a function of oxygen tension; previous paragraph, is dependent on numerous factors (e.g. species; donor age and/or gender; tissue origin; cell differentiation stage; media supplements; experimental conditions), imposing the need for standardisation.

## Conclusions

Herein, we assessed the influence of oxygen tension and macromolecular crowding in human bone marrow stem cell culture. Macromolecular crowding enhanced extracellular matrix deposition at both 20% and 2% oxygen tension. Interestingly, although the hypoxia inducible factor - 1*α* was activated at 2% oxygen tension, increased extracellular matrix synthesis was not observed. Neither surface markers nor transcription factors were affected as a function of oxygen tension and macromolecular crowding. With respect to the tri-lineage potential, adipogenesis was reduced in low oxygen tension cultures, chondrogenesis was increased in macromolecularly crowded cultures and osteogenesis was not affected as a function of oxygen tension and macromolecular crowding. Our data further corroborate the motion towards multi-factorial approaches for the development of physiologically relevant tissue-like supramolecular assemblies based on the principles of *in vitro* organogenesis.

## Additional Information

**How to cite this article**: Cigognini, D. *et al*. Macromolecular crowding meets oxygen tension in human mesenchymal stem cell culture - A step closer to physiologically relevant *in vitro* organogenesis. *Sci. Rep*. **6**, 30746; doi: 10.1038/srep30746 (2016).

## Supplementary Material

Supplementary Information

## Figures and Tables

**Figure 1:**
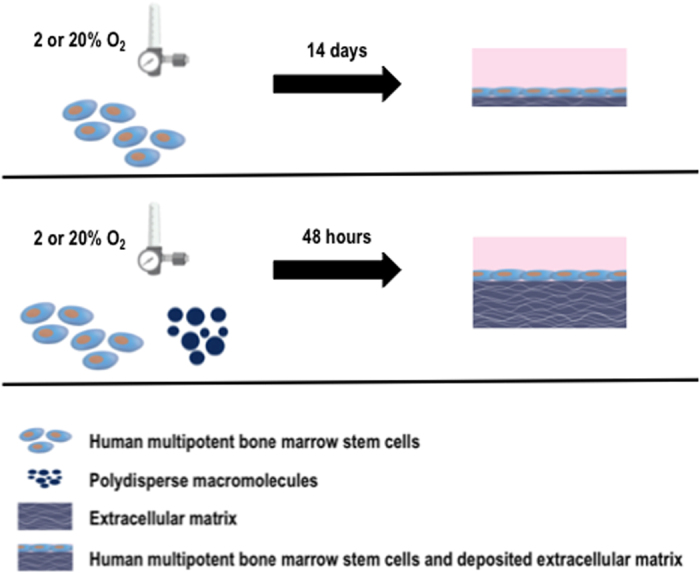
Macromolecular crowding significantly accelerates extracellular matrix deposition in human bone marrow mesenchymal stem cell culture at both 20% and 2% oxygen tension.
