# Supplementary material for: Macromolecular crowding meets oxygen tension in human mesenchymal stem cell culture - A step closer to physiologically relevant in vitro organogenesis
Source: Sci Rep. 2016 Aug 1;6:30746. doi: 10.1038/srep30746 (PMC4967872; doi:10.1038/srep30746)
Supplement: Supplementary Information [file srep30746-s1.pdf]

Supporting Information

Title

Macromolecular crowding meets oxygen tension in human mesenchymal stem cell culture - A step closer to physiologically relevant *in vitro* organogenesis

Authors

Daniela Cigognini (1, 2), Diana Gaspar (1, 2), Pramod Kumar (1, 2), Abhigyan Satyam (1, 2), Senthilkumar Alagesan (3), Clara Sanz-Nogués (3), Matthew Griffin (3), Timothy O'Brien (2, 3), Abhay Pandit (2), Dimitrios I. Zeugolis (1, 2)*

Affiliations

1. Regenerative, Modular & Developmental Engineering Laboratory (REMODEL), Biomedical Sciences Building, National University of Ireland Galway (NUI Galway), Galway, Ireland

2. Science Foundation Ireland (SFI) Centre for Research in Medical Devices (CÚRAM), Biomedical Sciences Building, NUI Galway, Galway, Ireland

3. Regenerative Medicine Institute (REMEDI), Biomedical Sciences Building, NUI Galway, Galway, Ireland

* Corresponding Author: Dr Dimitrios I. Zeugolis, REMODEL, NUI Galway, Galway, Ireland

Telephone: +353 (0) 9149 3166; Fax: +353 (0) 9156 3991; Email: dimitrios.zeugolis@nuigalway.ie

Methods

Human mesenchymal stem cell isolation from bone marrow: Bone marrow (Lonza) was obtained from 4 donors (3 females, 1 male; average donor age 23.3 ± 3.4). Samples were transferred into 50 ml tubes, washed with PBS and centrifuged at 900g for 10 min. Supernatant was aspirated without disturbing the pellet and mononucleated cells were seeded at a density of 2-3 x 10^5 cells/cm^2. Five days later, medium was replaced. Cells were fed twice per week and passaged when discrete colonies were formed (normally after 12-16 days). The identity of hBMSCs was confirmed using the following criteria: 1) adherence to plastic in standard culture conditions; 2) expression of CD105, CD73 and CD90 and lack of expression of CD3, CD14, CD19, CD34, CD45 and HLA-DR using BD FACS CANTO flow cytometer; and 3) ability to differentiate into osteoblasts, adipocytes and chondroblasts under standard *in vitro* differentiation conditions, as demonstrated by staining for calcium deposits, lipid droplets and sulphated GAGs, respectively.

Phase contrast microscopy: Cell morphology was evaluated at the different time points to evaluate the influence of crowding. Images were taken using an inverted microscope (Leica).

Assessment of cell metabolic activity: Cells were washed with Hanks' Balanced Salt solution (HBSS), then alamarBlue® (Invitrogen) was added according to the manufacturer's protocol. After 4 hours of incubation at 37 ^{o}C, absorbance was measured at 550 nm and 595 nm using Varioskan Flash spectral scanning multimode reader (Thermo Scientific). Cell metabolic activity was expressed as % difference in reduction of alamarBlue® between MMC-treated cells and untreated cells at day 2.

Assessment of cell viability: Cell viability was evaluated using the CytoTox 96® Non-Radioactive Cytotoxicity Assay (Promega), following manufacturer's instructions. Conditioned media were collected and incubated with the Substrate Mix at room temperature. After 30 minutes, absorbance

was measured at 490 nm in a plate reader (Varioskan Flash, Thermo Scientific). Cell viability was expressed as % difference between MMC-treated cells and untreated cells at day 2.

Tri-lineage differentiation assay: Adipogenic, osteogenic and chondrogenic assays were performed in technical triplicates in one experiment. Cells were cultured either with crowded medium containing 100 μg/ml carrageenan or with CTR medium, at either 20% or 2% O_2. After two weeks, cells cultured under the four conditions (CTR 20% O_2, CTR 2% O_2, C100 20% O_2 and C100 20% O_2) were harvested and fed with differentiating media at 20% O_2. Differentiation ability was determined by histological and biochemical analysis. For each assay, negative controls were fed with complete medium. Adipogenic, osteogenic and chondrogenic differentiation were induced (BulletKit™, Lonza) according to manufacturer's protocol. Adipogenic differentiation was evaluated by Oil Red O staining and uptake quantification. Osteogenic differentiation was assessed by Alizarin Red S staining and uptake quantification. Chondrogenic differentiation was analysed by Safranin O / Fast Green staining and GAG quantification using 1,9-dimethylmethylene blue (DMMB) method. GAG content of pellets was normalised to DNA amount and expressed as μg per μg DNA.

Results

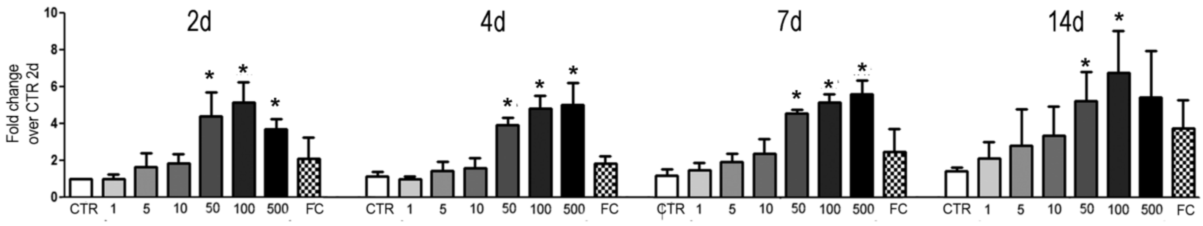

Figure S1: Complementary densitometric analysis of SDS-PAGE of Figure 1 demonstrates enhanced ECM deposition under MMC conditions. 100 μg/ml CR appears to be the minimum effective concentration for maximum ECM deposition. *: Statistically significant different from the CTR at a given time point. Note: Densitometric analysis of the α1 and α2 bands was performed with ImageJ software (NIH).

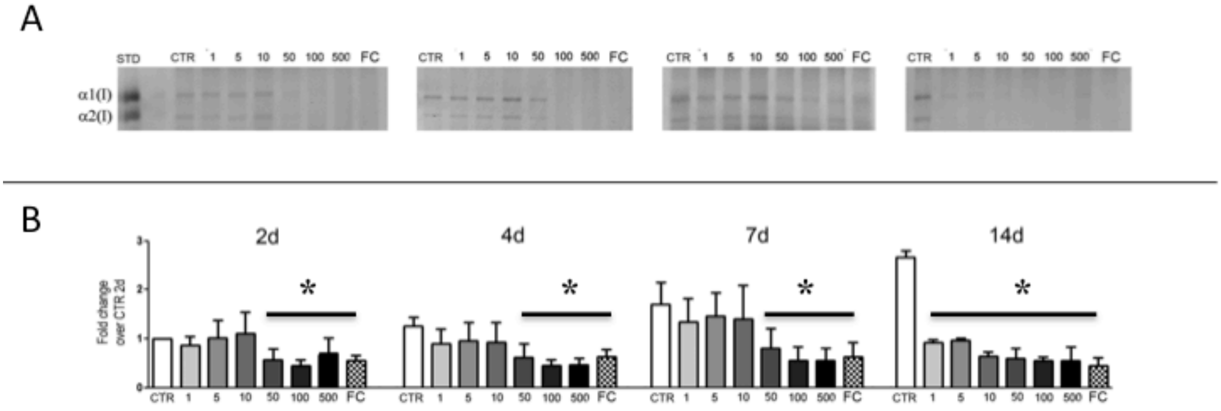

Figure S2: SDS-PAGE (A) and complementary densitometric (B) analyses demonstrate reduction in collagen type I content in the media, as a function of increased CR concentration. *: Statistically significant different from the CTR at a given time point. Note: Densitometric analysis of the $\alpha 1$ and $\alpha 2$ bands was performed with ImageJ software (NIH).

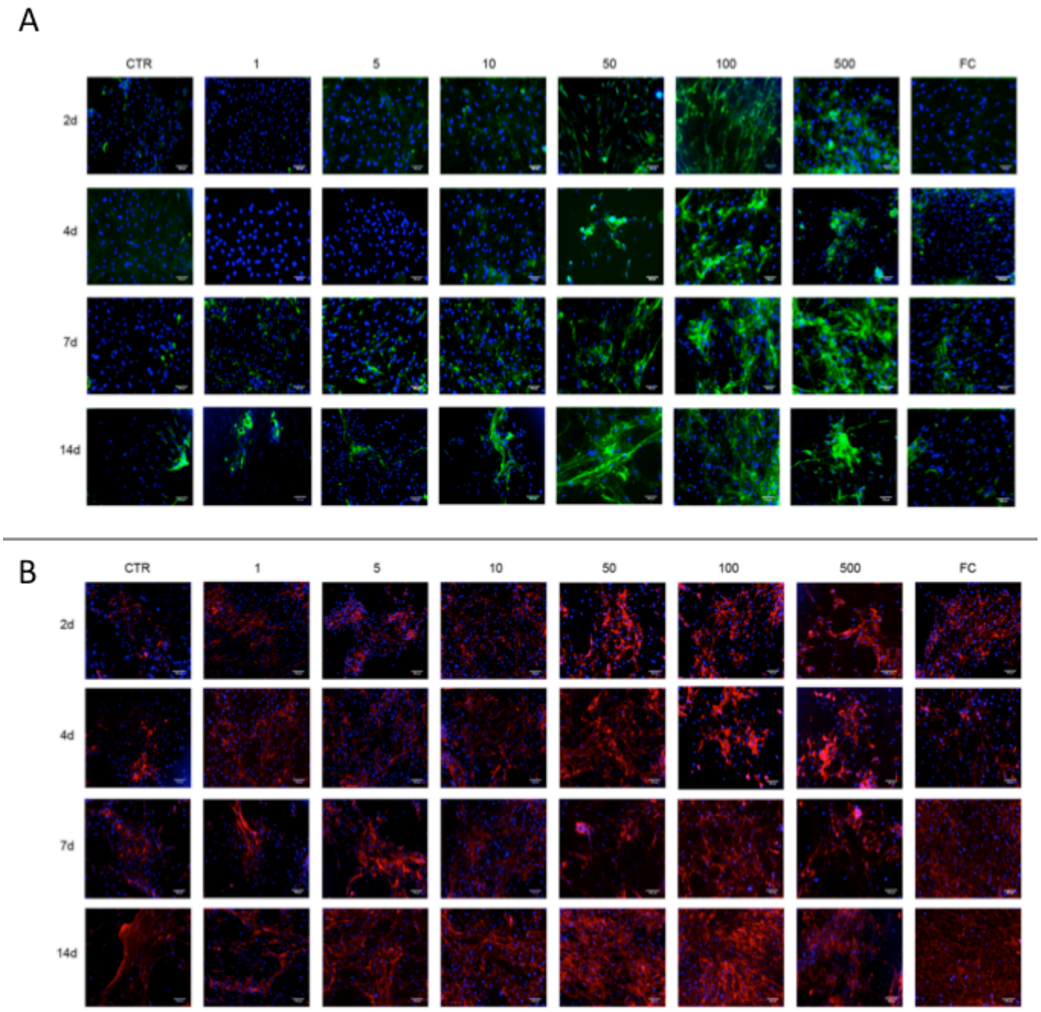

Figure S3: Immunocytochemistry analysis indicates enhanced collagen type I (A) and collagen type III (B) deposition in the presence of 100 and 500 μg/ml CR after 4, 7 and 14 days in culture.

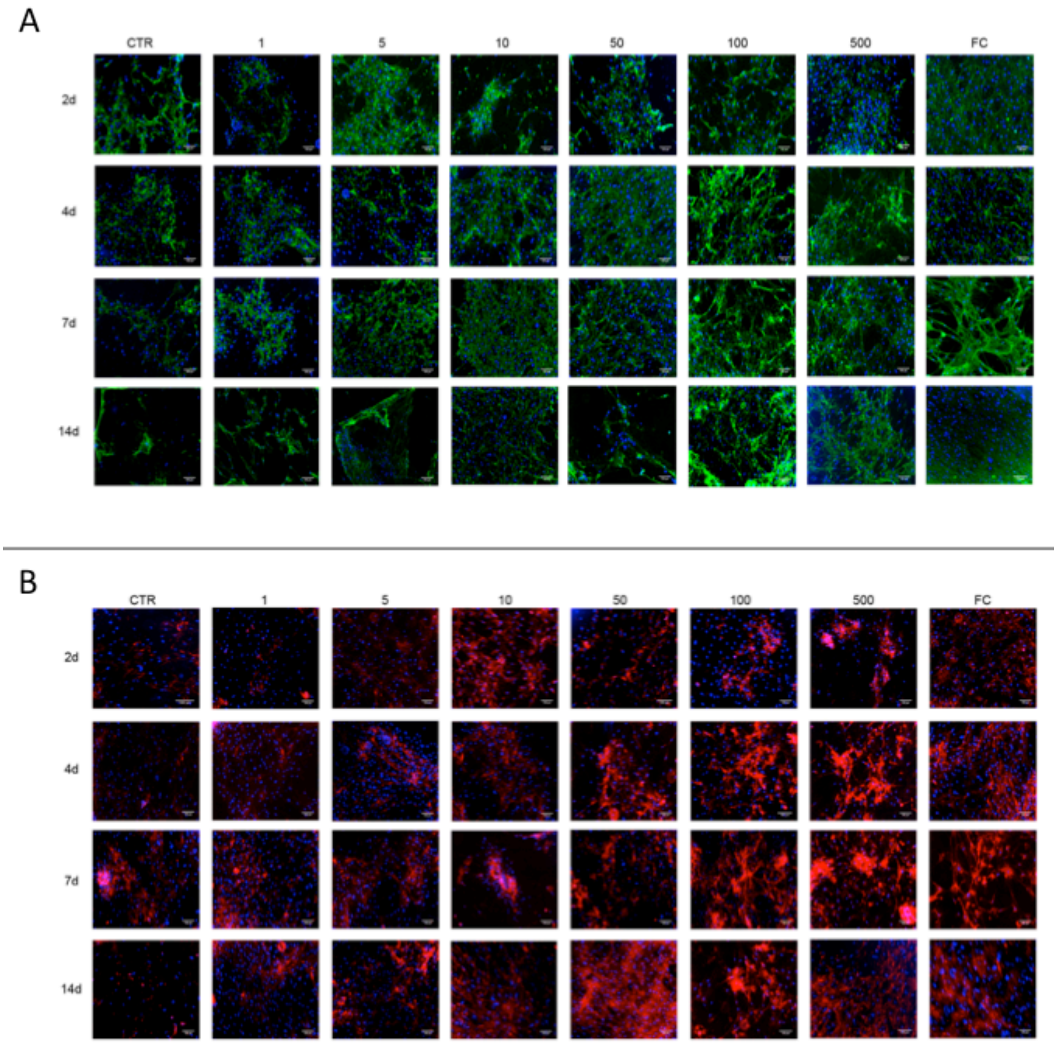

Figure S4: Immunocytochemistry analysis indicates enhanced fibronectin (A) and laminin (B) deposition in the presence of 100 and 500 μg/ml CR after 4, 7 and 14 days in culture.

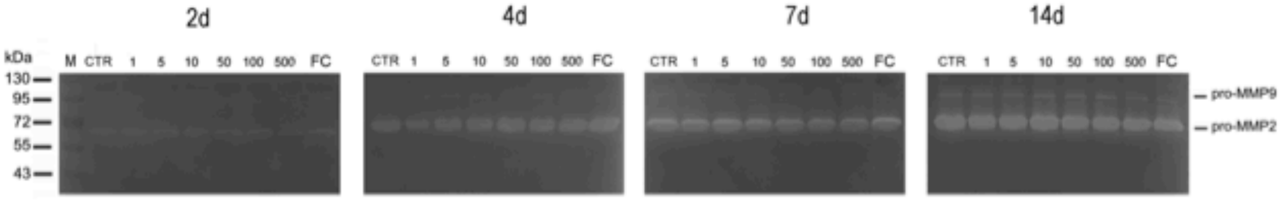

Figure S5: Gelatin zymography of the media reveals an increased secretion of pro-MMP2 and pro-MMP9 as a function of time in culture.

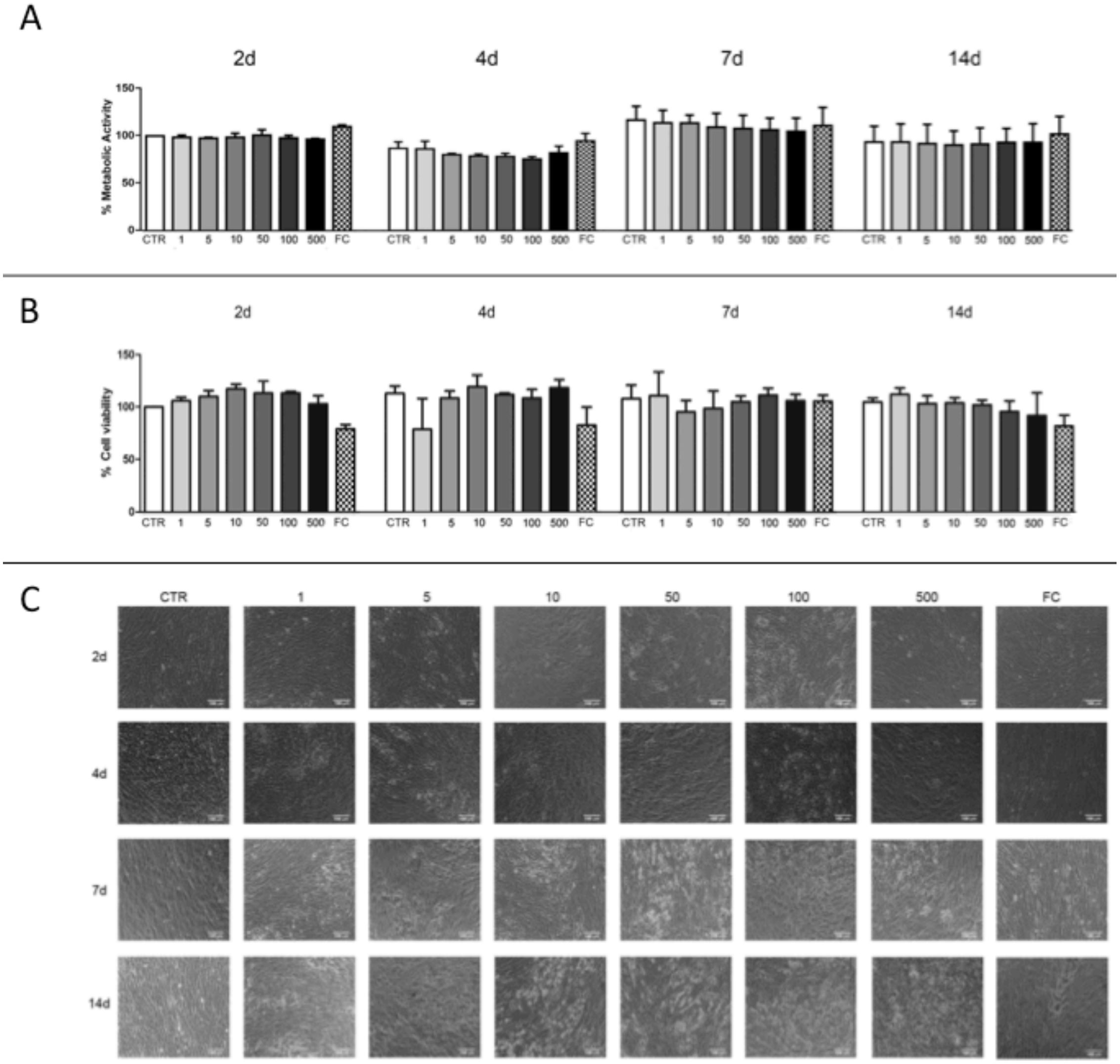

Figure S6: Cell metabolic activity (A), viability (B) and morphology (C) are not affected as a function of treatment and time in culture.

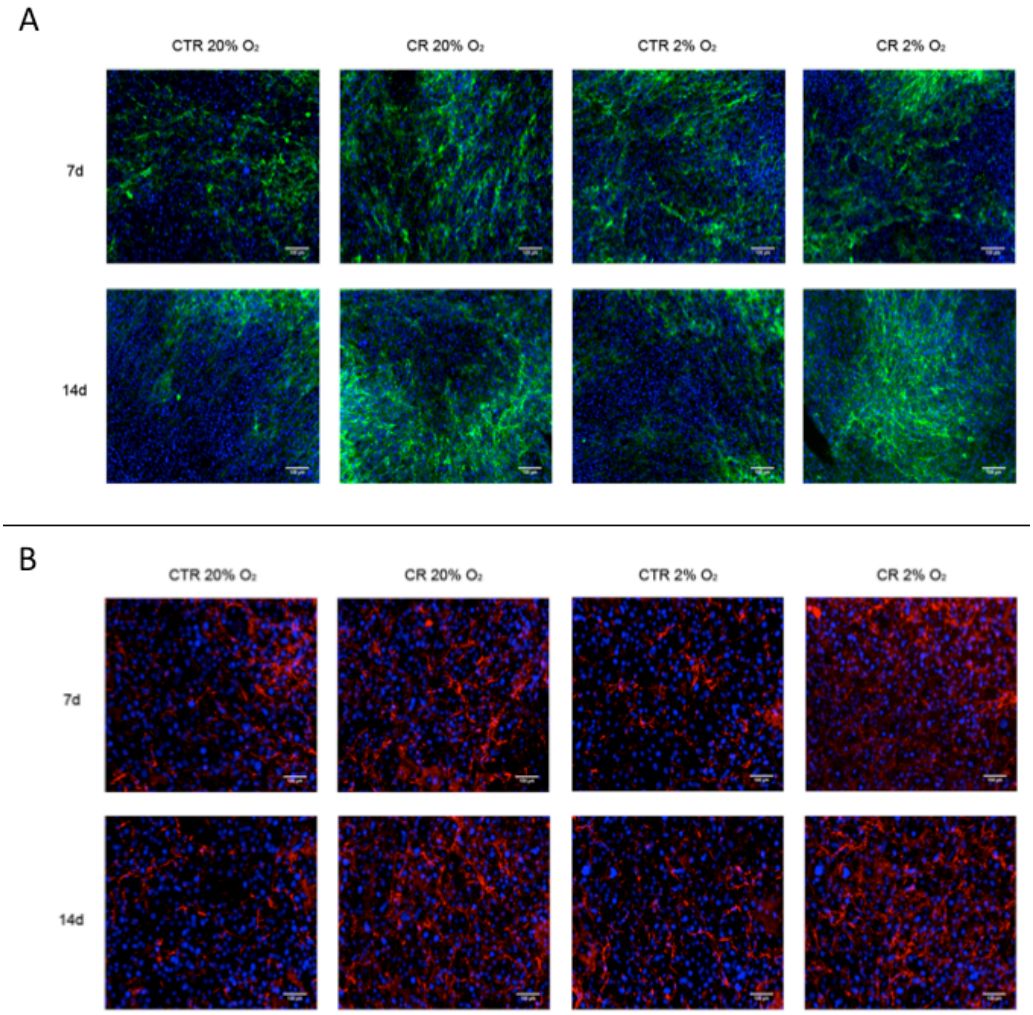

Figure S7: Immunocytochemistry analysis indicates increased collagen type I (A) and collagen type III (B) deposition under MMC conditions at both 20% and 2% oxygen tension.

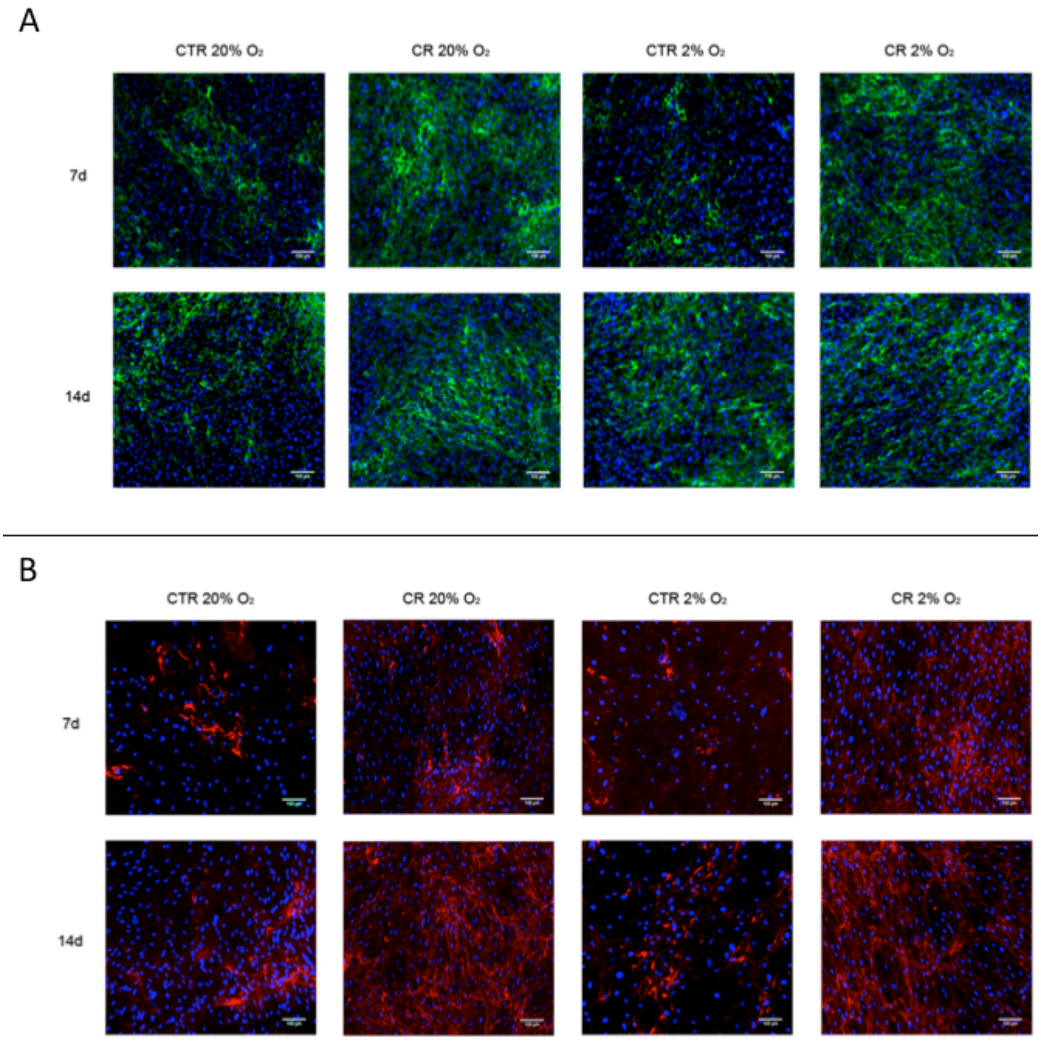

Figure S8: Immunocytochemistry analysis indicates increased fibronectin (A) and laminin (B) deposition under MMC conditions at both 20% and 2% oxygen tension.

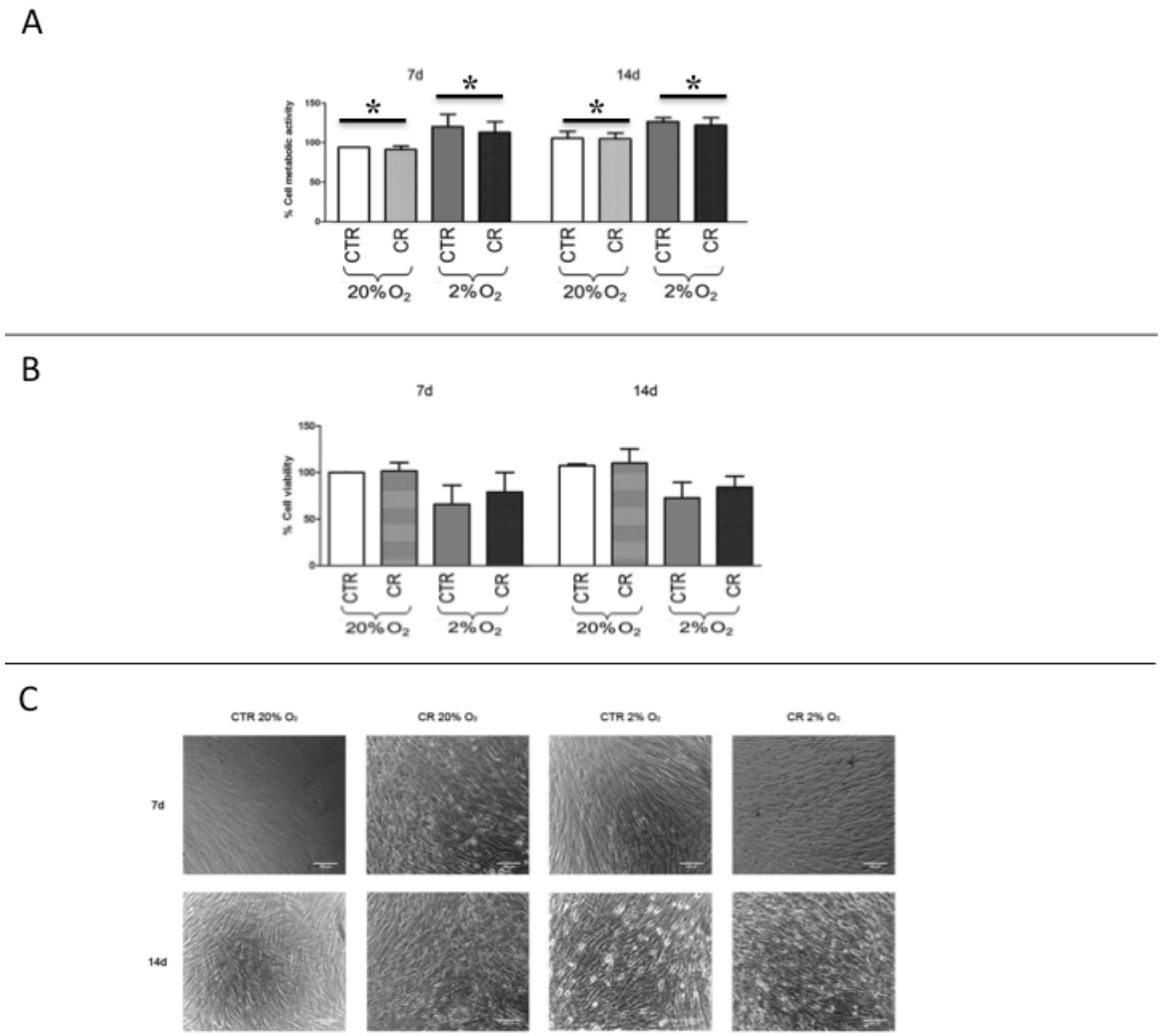

Figure S9: Cell metabolic activity (A) is significantly enhanced at 2% oxygen tension. Cell viability (B) and morphology (C) are not affected as a function of oxygen tension (20% and 2%) and MMC. *: Statistically significant different.

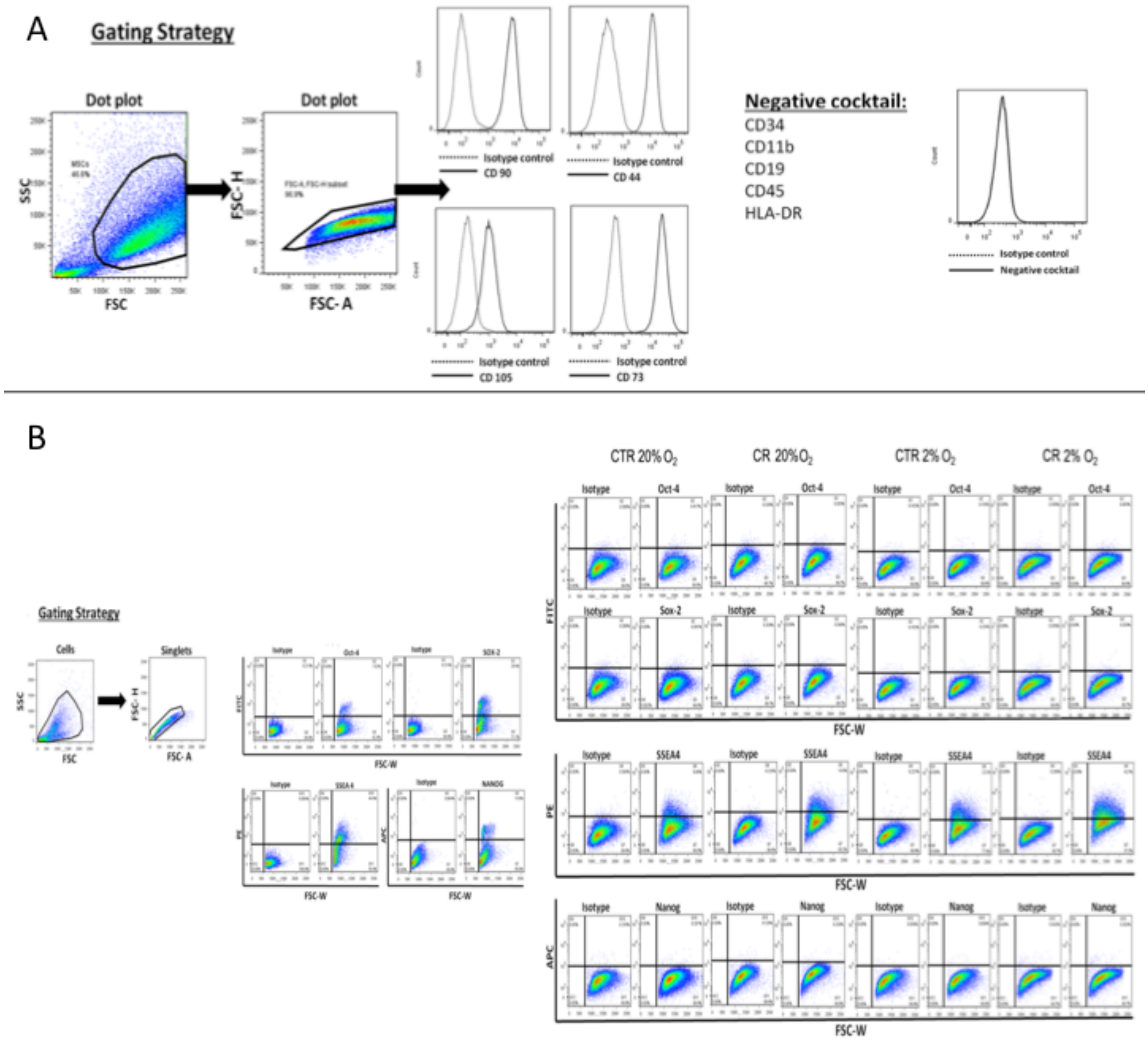

Figure S10: (A) hBMSCs were stained with CD90, CD44, CD105 or CD73 and ~30,000 events were recorded in the BD FACSCanto® cytometer. Live cells were gated out of debris and dead cells and then doublet cells were excluded. Only single cells were included for the cell surface marker expression. Median Fluorescence Intensity (MFI) was calculated and histograms were plotted by FlowJo® software (version 7). hBMSCs were also stained for negative markers CD34, CD11b, CD19, CD45 and HLA-DR, which showed lack of expression in all experiments. These data indicate that cells used for the experiments came from homogenous cultures of hBMSCs. (B) Staining for Oct-4, Sox2, SSEA-4 and Nanog was optimised on induced pluripotent stem cells (iPSCs), which stained positive for all markers. Representation of dot plots from intracellular staining performed on hBMSCs.

Table S1: Primers for real-time PCR.

Gene name (protein name)	Primer sequence 5' > 3'	Product length (bp)
ACTB (beta-actin)	F: CCCAGATCATGTTTGAGACCT<br>R: GAGTCCATCACGATGCCAGT	106
PPIA (cyclophilin A)	F: GTCAACCCCACCGTGTTCTTC<br>R: TTTCTGCTGTCTTTGGGACCTTG	100
NANOG (Nanog)	F: AACTCTCCAACATCCTGAACC<br>R: CCTTCTGCGTCACACCATT	126
POU5F1 (Oct-4)	F: GTTGGAGGGAAGGTGAAGTTC<br>R: TGTGTCTATCTACTGTGTCCCA	103
SOX2 (Sox2)	F: GTACAACTCCATGACCAGCTC<br>R: CTTGACCACCGAACCCAT	118
ST3GAL2 (SSEA-4 synthase)	F: TGGACGGGCACAACTTCATC<br>R: GGGCAGGTTCTTGGCACTCT	119
COL1A1 (collagen I, alpha 1)	F: CCTGGATGCCATCAAAGTCT<br>R: CGCCATACTCGAACTGGAAT	170
P4HA1 (prolyl 4-hydroxylase, alpha I)	F: TGATGTGTCTGCAGGAGGAG<br>R: TTGTTGCCAACTAGCACTGG	156
